# Single mandibular implant study - impact on dietary habits after 5 years of observation in patients with immediate and delayed loading protocols

**DOI:** 10.1007/s00784-024-05970-2

**Published:** 2024-10-04

**Authors:** Sarah M. Blender, Christoph Behrendt, Elfriede Fritzer, Stefanie Kappel, Ralf J. Kohal, Ralph G. Luthardt, Nadine Frfr. v. Maltzahn, Daniel R. Reissmann, Stefan Wolfart, Matthias Kern, Nicole Passia

**Affiliations:** 1 Center of Dentistry, Department of Prosthetic Dentistry, Ulm University Hospital, Ulm, Germany; 2 Department of Prosthodontics, Gerodontology and Biomaterials, Greifswald University Hospital, Greifswald, Germany; 3 Institute of Medical Informatics and Statistics, Center for Clinical Studies, Christian-Albrechts University at Kiel, Kiel, Germany; 4 Department of Prosthodontics, Heidelberg University Hospital, Heidelberg, Germany; 5 Department of Prosthetic Dentistry, Center for Dental and Oral Medicine, University Medical Center Freiburg, Freiburg, Germany; 6 Department of Prosthodontics, University Hospital Hannover, Hannover, Germany; 7 Department of Prosthetic Dentistry, Center for Dental and Oral Medicine, University Medical Center Hamburg-Eppendorf, Hamburg, Germany; 8 Present Address: Department of Prosthodontics and Material Sciences, University of Leipzig, Leipzig, Germany; 9 Department of Prosthodontics and Biomaterials, Medical Faculty, RWTH Aachen University, Aachen, Germany; 10 Department of Prosthodontics, Propaedeutics and Dental Materials, School of Dentistry, Christian-Albrechts University at Kiel, Kiel, Germany; 11 Department of Prosthodontics, Faculty of Medicine and University Hospital Carl Gustav Carus, Technische Universität Dresden, Dresden, Germany

**Keywords:** Single mandibular implant, Loading protocol, Overdenture, Dietary habit, Nutrition intake

## Abstract

**Objectives:**

Single midline implants in the edentulous mandible can be used to support existing complete dentures to improve patients’ satisfaction and masticatory efficiency. The impact on patients’ dietary habits and the influence of the loading protocol of the implants was the subject of this study.

**Materials and methods:**

In this prospective randomized clinical trial, edentulous patients with existing complete dentures in both jaws were treated with a single midline implant in the mandible. In group A, the implants were loaded immediately, in group B the loading was delayed after three months. Patients were asked to report on their nutritional intake before implant placement and 12, 24 and 60 months after loading using a standardized two-part questionnaire.

**Results:**

Nutritional intake regarding the frequency of consumption of the requested food items did not change significantly during the 60-months study period, regardless of the loading protocol. In contrast, the second part of the questionnaire revealed that after 60 months, there was a significant decrease in avoidance of food, that had a coarse and hard texture in both groups. This significant decrease was observable in the group A in the first 12 and 24 months and in the group B after 60 months.

**Conclusion:**

A change in the patients’ dietary habits due to the insertion of a single midline implant in the mandible to support the existing complete denture cannot be observed, independently to the loading protocol.

**Clinical relevance:**

Improving the chewing efficiency by single midline implants in the edentulous mandible does not lead to a change in dietary habits.

## Introduction

For many years, conventional complete dentures were considered the treatment of choice for the rehabilitation of the esthetic and functional components of edentulous patients [1, 2]. The retention and stability of these dentures is highly influenced by the existing anatomical supporting tissue, the adjusted tooth set-up and a targeted extension of the denture base into the muscular functional spaces [3, 4]. Due to atrophy of the mandible and reduced supporting tissue compared to the maxilla, there are limits regarding a sufficient retention and stability of complete dentures in the mandible, which can directly influence dietary habits and patient comfort [5–7].

In addition to patients’ satisfaction and masticatory efficiency, the use of oral implants can also increase the stability of these prostheses [8]. As early as 2002, the Mc Gill Consensus Statement set implant-supported complete dentures on two implants to be the standard and first-choice therapy for the treatment of edentulous mandibles [9]. For prosthetic and anatomical reasons, the intraforaminal region is an excellent position for a simple implant insertion. The absence of a nerve canal and adequate vertical residual bone height even in advanced atrophy are advantage [10, 11]. Suitable retention elements such as ball anchors, bars and magnets can be used to allow direct attachment to the prosthesis [12, 13]. In some cases, existing prostheses can be modified in this process, even avoiding the need to fabricate a new prosthetic restoration.

A comparison of conventional complete dentures with implant-supported dentures on two implants shows a significant improvement in patients’ comfort, oral health-related quality of life, denture stability, phonetics and, finally, increased masticatory efficiency [8, 14–16]. The positive influence of the use of implants was also observed when comparing conventional complete dentures with centrally inserted implants in the lower jaw to support a complete denture [17, 18]. Although this has improved the conditions for a more multifaceted nutritional intake, the literature does not demonstrate a significant change in dietary habits in this context [19, 20].

Depending on the time after implant placement, the implants can be loaded immediately or with a delayed loading. Loading is defined as the direct occlusal contact of the implant-supported denture with the antagonistic opposite dentition. Immediate loading requires an inflammation-free and sufficient implant area, controlled occlusal loading and, of course, adequate primary stability of the implants [21]. Specifically for the placement of implants in the mandible, these indications are sufficiently given, even in the case of advanced atrophy [22]. With this concept, patients’ expectations of the new prosthesis in regard to comfort and improved chewing efficiency can be realized immediately after surgery [23]. In this context, studies do not demonstrate an increased loss of marginal bone with immediate loading of implant-supported prostheses on two implants in the mandible [24, 25]. However, when considering the risk of implant loss, there is a trend towards possible early loss of implants within the first few months if they are loaded immediately compared to delayed loading [26, 27].

The retention of complete dentures using only one centrally placed implant in the mandible has also been investigated and demonstrated similar improvement performance to implant-supported dentures on two implants [28, 29]. For a therapeutic decision concerning the loading protocol, the risks of immediate loading should be evaluated against its benefits. If necessary, an open healing with moderate loading should be attempted [30]. Similar to the investigations with two implants, studies revealed an improvement in patient satisfaction, oral health-related quality of life and patient comfort [31–33]. In addition, a significant improvement in masticatory efficiency can be observed here. Regardless of the loading protocol, an initial significant increase in chewing efficiency was observed in both study groups in the present study population, which is still existent after 60 months [34]. However, whether and to what extent this form of therapy has an influence on the patients’ nutritional intake has not been investigated yet.

The aim of this study was to investigate the influence of the loading protocol of the implants on a possible change in the dietary habits of patients after a midline implant was inserted in the mandible to support the existing complete denture.

## Materials and methods

The present study is a prospective randomized clinical trial conducted at nine study centers in Germany. The study was registered at the German register of clinical trials (Deutsches Register Klinischer Studien; DRKS-ID: DRKS00003730) and the study design was approved by the Institutional Review Boards of the participating centers (approval number of the leading study center at Kiel University: A 138/12). To guarantee a calibrated performance of each center, standardized protocols were established for each step and a coordination meeting was held. Written informed consent was obtained from all patients recruited into the study before possible inclusion; this was repeated and updated again for the 5-year follow-up.

The results of the present study were obtained from a patient pool of a multicenter study over several years. Various publications with different investigation outcomes have already been published [34–43]. This publication is the first to analyze a possible influence on the dietary habits of these patients with an overdenture on a midline implant in the mandible for immediate or delayed loading.

In the present study, edentulous patients between 60 and 89 years with sufficient and technically acceptable complete dentures in the maxilla and mandible were included, whereby the retention and/or stability of the mandibular denture was assessed as insufficient by the patients. If a new mandibular prosthesis was fabricated, it had to be in situ for at least 3 months to allow adaptation by the patient. For the placement of a single midline implant in the mandible, the residual bone height at the lowest vertical height of the mandible had to range from 11 to 20 mm (according to Mc Garry et al. type II or type III [44]) and the vertical bone height at the midline of the mandible had to measure at least 13 mm.

A detailed overview of the inclusion and exclusion criteria, as well as the detailed study protocol and CONSORT flowchart, has been described in previous publications [35, 36, 42].

After completion of the screening of potential study patients, a total of 163 patients received a single midline mandible implant (3.8 × 11 mm; Promote plus, Camlog Biotechnologies, Basel, Switzerland). Of these, 158 patients could be randomized into the two study groups. Randomization took place at the time of implantation directly after insertion of the implant and determination of the implant stability (insertion torque/ISQ value). The insertion torque had to be at least 30 Ncm and the ISQ value had to be ≥ 60. To determine the ISQ value, a Smart Peg attachment (type 23) was screwed hand-tight onto the implant (2–4 Ncm) and the implant stability was measured in mesio-distal and vestibular-oral direction using resonance frequency analysis (Osstell ISQ, Gothenburg, Sweden). The implants of patients in group A (*n* = 81) were loaded immediately, whereas in group B (*n* = 77) a delayed loading was performed after a defined healing period of 3 months. For fixation of the prosthesis with the ball attachments (Dalbo-Plus Elliptic, Cendres Métaux, Biel, Switzerland) on the implants, the retention matrices were inserted into the existing mandibular prosthesis with a self-curing bis-acrylate resin (LuxaPick-up, DMG, Hamburg, Germany), using the chair-side technique.

### Dietary questionnaire

In order to assess the influence of the implant loading protocol on changes in nutrition intake over time, a corresponding dietary questionnaire was created for the study, adapted and based on Thiel et al. [45] and Quandt et al. [46]. This questionnaire was completed by the patients in both study groups at four defined time points: (1) baseline examination before randomization and implantation, (2) 12 months after implant loading, (3) 24 months after implant loading and (4) 60 months after implant loading.

The first part of the dietary questionnaire was aimed at the patients’ nutrition intake. The patients were asked to document how frequently certain foods were consumed. Nine foods and three alcoholic beverages were recorded. There were seven possible answers ranging from “1 - rarely or never” to “7 - two or more times a day”. According to the consumption frequency, the patient could enter the corresponding answer option with a number behind each food. Table 1 provides an overview of the foods queried.

**Table 1:** Part 1 of the dietary questionnaire: query of the frequency of intake of certain foods and beverages by the patients. The corresponding number (1–7) indicated the frequency

How frequently do you consume the following foods and beverages?	Options for response
Beef	
Pork	
Poultry	1: rarely or never
Leafy vegetables, raw or cooked	2: 1–2 times per month
Red or yellow vegetables, raw or boiled	3: 1–2 times per 14 days
Raw vegetables	4: 1–3 times per week
Fresh fruits	5: 3–6 times per week
Unprocessed grains (brown rice, cereal, flaked oats, etc.)	6: once a day
Whole grain bread	7: twice or several times a day
Alcoholic beverages: beer	
Alcoholic beverages: wine	
Alcoholic beverages: liquor	

In the second part of the nutrition questionnaire, the patients were asked whether they actively avoided certain foods as a result of the existing dentures. Eleven foods with different consistencies and textures were submitted to the patients. This time, the patients had to specify whether they consciously actively avoid these foods in their diet due to the presence of the denture. Simple “yes” or “no” answers were possible (Table 2).

**Table 2:** Part 2 of the dietary questionnaire: query on the active avoidance of certain foods due to the denture by the patients. These questions could be answered with “yes” or “no”

Are you avoiding any of the following foods?	Options for response
Apples with peel	
Whole nuts, not chopped	
Sticky candy	
Raw carrots	
Chops or steaks	“Yes” or “No”
Lettuce	
Berries (strawberries, raspberries, blueberries, etc.)	
Grilled chicken	
Raw tomatoes	
Fried fish	
Dark bread	

### Statistical analysis

In the statistical analysis of the five-year data, only subjects were included for whom a completely answered nutritional questionnaire was available for all four examination dates.

The comparison of the two groups A and B regarding the consumption frequency of certain foods at the corresponding times was performed using the non-parametric Wilcoxon rank sum test. The change in consumption frequency over time was examined within a group using the Friedman-test. For the subsequent pairwise comparisons of the individual time points within a group, the Wilcoxon sign rank test was used. For comparison of the two groups in active avoidance of certain foods, Fisher’s exact test was used for intergroup comparisons at each times and McNemar’s test was used for intragroup comparisons between two study time points. Changes over time within groups were examined using the Cochran-Q-test. The significance level was set at *p* ≤ 0.05 for all tests. No correction of the p-value for multiple testing was performed, as this was an exploratory analysis of the data obtained.

## Results

During the five-year follow-up, 50 patients in group A and 51 patients in group B were re-examined, with one patient in group B having an implant loss. The number of patients in group A and group B was reduced to 47 and 45, respectively, since only for those patients dietary questionnaire data were available at all four examination times. Table 3 provides an overview of the characteristics of the study population included in the evaluation described here at time of screening.

**Table 3:** Summary of patient characteristics at the time of patient screening

	Total number of patients		Group A		Group B	
Number of patients	92		47		45	
Age in years (± standard deviation)	69.3 ± 5.9		70.4 ± 6.3		68.2 ± 5.3	
	N	%	N	%	N	%
female	50	54.3%	27	57.4%	23	51.5%
male	42	45.7%	20	42.6%	22	48.9%

### Dietary questionnaire part 1: intra- and intergroup changes in the frequency of consumption of certain foods

**Fig. 1:**
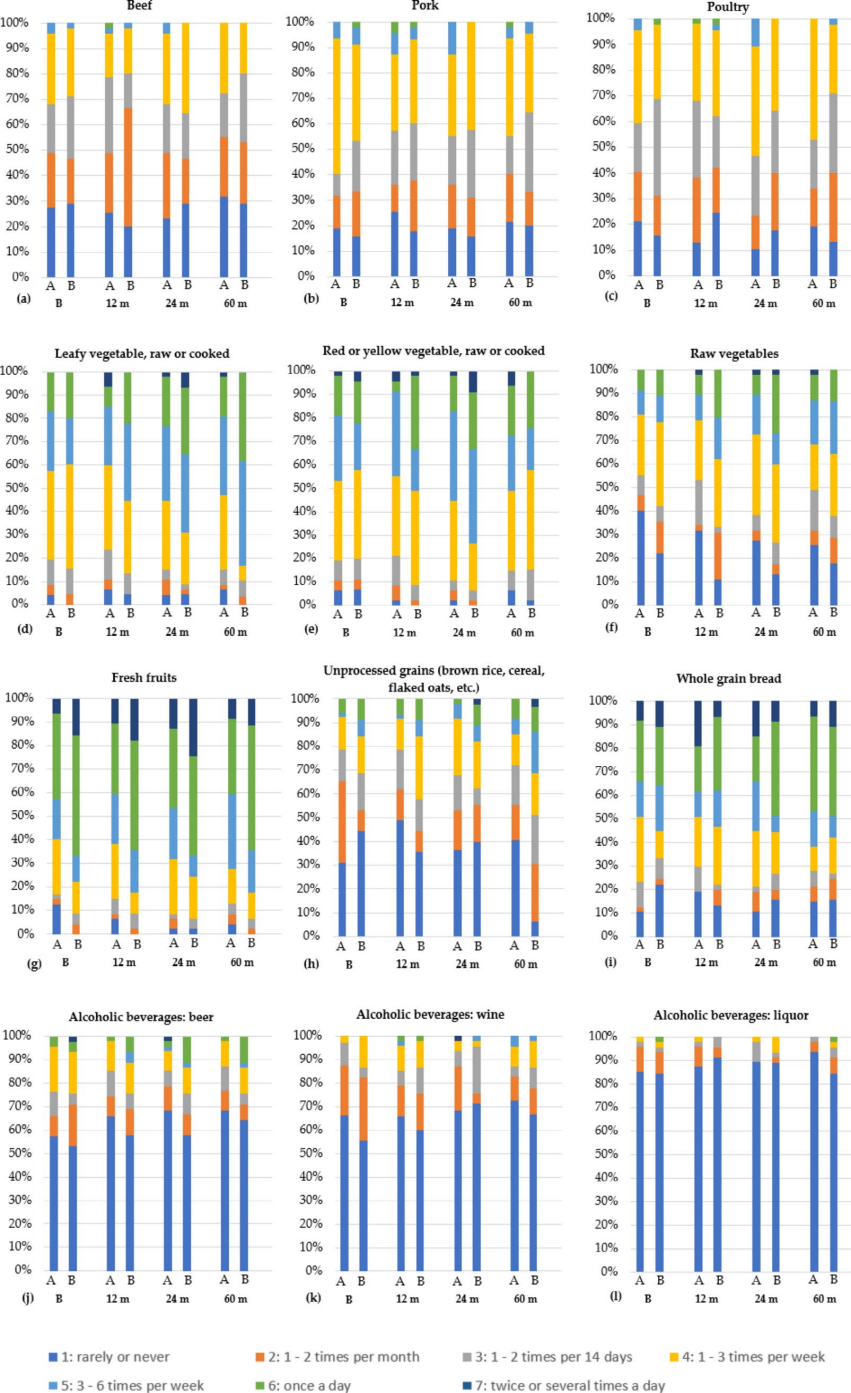
**(a-l).** Figure 1 shows the consumption frequency of the two study groups A and B regarding each food (**a-l**) during the examination dates (B) Baseline, (12 m) 12 months after loading, (24 m) 24 months after loading and (60 m) 60 months after loading

Figure 1 (a-l) maps the consumption frequencies of groups A and B at the different examination dates for the nine queried foods and three alcoholic beverages. For the foods beef, pork, unprocessed cereals (brown rice, cereal, oatmeal, etc.), whole-grain bread and the alcoholic beverages beer, wine, and liquor, neither intergroup nor intragroup differences were observed throughout the study period. At baseline, only the consumption of fresh fruit showed a significant difference between the food intake of the two groups (*p* = 0.013). This trend was also observed at 12-month (*p* = 0.020) and 60-month (*p* = 0.045) follow-up, while there were no significant intragroup changes in food intake over time in either group. In this context, group B showed an increased intake of fresh fruit at baseline and at all significant follow-up periods.

For poultry, a significant difference was observed between the two groups after 24 months of loading (A < B; *p* = 0.031). Thereby, a significant increase in the consumption of poultry (*p* = 0.003) was observed in group A when comparing the baseline examination with the examination after 24 months after loading. For red and yellow vegetables (raw or cooked), a significant difference between the groups was recorded after 24 months (A < B; *p* = 0.032). Here, the consumption habits in group B changed significantly over the course of the study (*p* = 0.002), whereby the comparison between the baseline examination and the follow-up examination after 24 months was significant (*p* = 0.002). The consumption of raw food 12 months after loading demonstrated a significant difference between the two groups (A < B; *p* = 0.042). The time course within group A showed significant differences in comparison (*p* = 0.047). Thereby, the consumption behavior of raw food in group A increased significantly not only after 12 months (*p* = 0.016), but as well after 24 months (*p* = 0.022). In addition, a significant increase in the consumption behavior of raw food in group B was observed after 24 months (*p* = 0.009). Although no differences in the consumption of leafy vegetables (raw or cooked) were observed between the groups, at least the time course in group B showed a significant change (*p* = 0.005): In this case, the frequency of consumption showed a significant increase of consumption between the baseline examination and the examination after 24 months (*p* = 0.019).

Differences related to the consumption of the food described when comparing the two groups at each time are shown in Table 4.

**Table 4:** Comparison of the two study groups for differences in consumption frequency of the recorded foods at each examination date using the Wilcoxon rank sum test (*p* ≤ 0.05; no correction of the p-value for multiple testing was performed); * *p* > 0.05

Foods	Baseline	12 months after loading	24 months after loading	60 months after loading
Beef	*	*	*	*
Pork	*	*	*	*
Poultry	*	*	*p* = 0.031	*
Leafy vegetables, raw or cooked	*	*	*	*
Red or yellow vegetables, raw or boiled	*	*	*p* = 0.032	*
Raw vegetables	*	*p* = 0.042	*	*
Fresh fruits	*p* = 0.013	*p* = 0.020	*	*p* = 0.045
Unprocessed grains (brown rice, cereal, flaked oats, etc.)	*	*	*	*
Whole grain bread	*	*	*	*
Alcoholic beverages: beer	*	*	*	*
Alcoholic beverages: wine	*	*	*	*
Alcoholic beverages: liquor	*	*	*	*

A detailed overview regarding the comparison of the time course within the two groups, as well as the pairwise comparison of the baseline examination to the different follow-up examinations (12, 24 and 60 months after loading) in group A and B are shown in Table 5.

**Table 5:** Comparison of time course regarding the frequency of food consumption within the two groups a and B for differences using the Friedman-test (*p* ≤ 0.05; no correction of the p-value for multiple testing was performed). Pairwise comparison of the baseline investigation to each study times for groups a and B using the Wilcoxon signed-rank test. (*p* ≤ 0.05; no correction of the p-value for multiple testing was performed); * *p* > 0.05

Foods	Comparison of time course regarding the frequency of food consumption		Baseline vs. 12 months after loading		Baseline vs. 24 months after loading		Baseline vs. 60 months after loading	
	Group A	Group B	Group A	Group B	Group A	Group B	Group A	Group B
Beef	*	*	*	*	*	*	*	*
Pork	*	*	*	*	*	*	*	*
Poultry	*	*	*	*	*p* = 0.003	*	*	*
Leafy vegetables, raw or cooked	*	*p* = 0.005	*	*	*	*p* = 0.019	*	*
Red or yellow vegetables, raw or boiled	*	*p* = 0.002	*	*	*	*p* = 0.002	*	*
Raw vegetables	*p* = 0.047	*	*	*	*p* = 0.016	*p* = 0.009	*p* = 0.022	*
Fresh fruits	*	*	*	*	*	*	*	*
Unprocessed grains (brown rice, cereal, flaked oats, etc.)	*	*	*	*	*	*	*	*
Whole grain bread	*	*	*	*	*	*	*	*
Alcoholic beverages: beer	*	*	*	*	*	*	*	*
Alcoholic beverages: wine	*	*	*	*	*	*	*	*
Alcoholic beverages: liquor	*	*	*	*	*	*	*	*

### Dietary questionnaire part 2: inter- and intragroup differences in the avoidance of certain foods

**Fig. 2:**
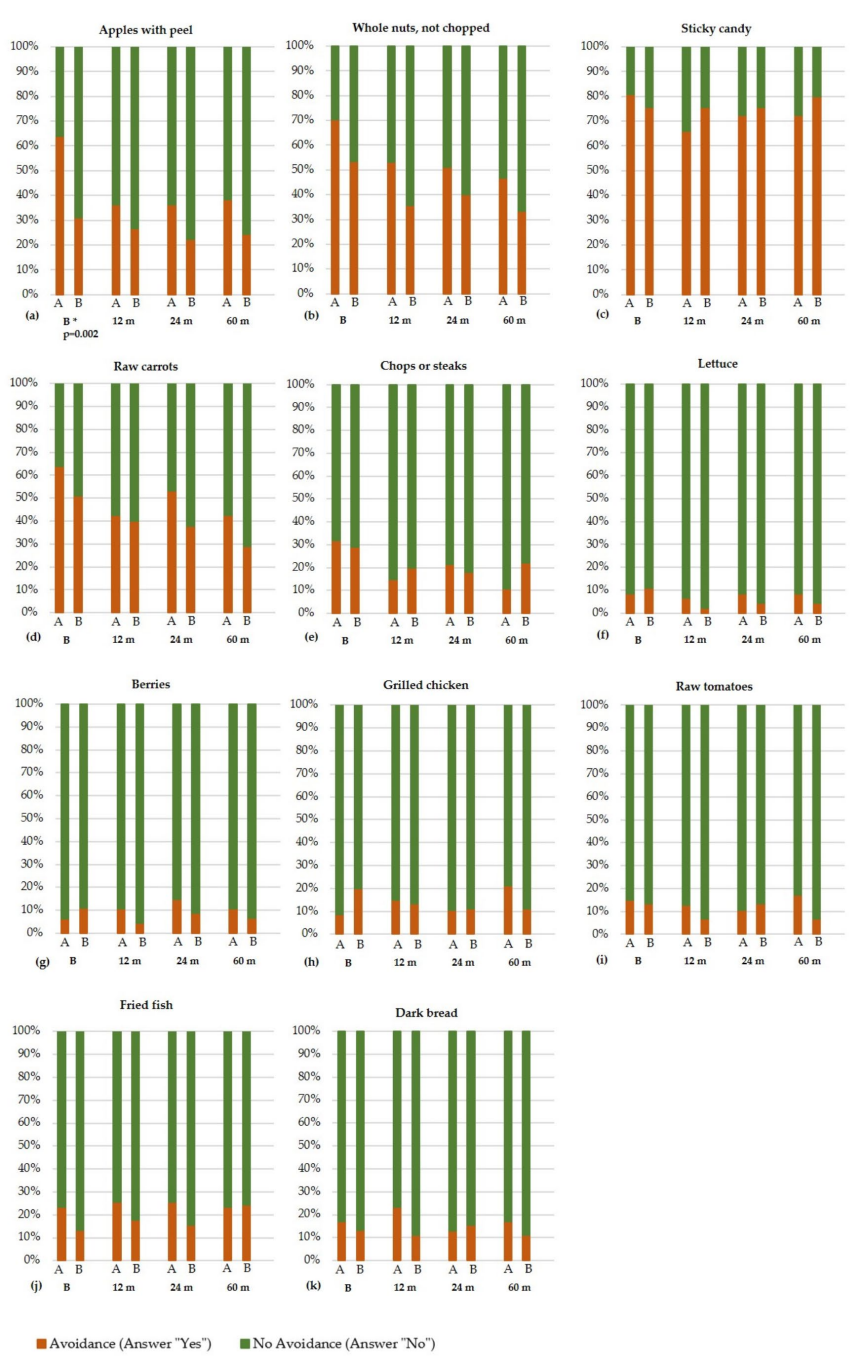
**(a-k).** Overview of the foods in the second part of the dietary questionnaire, asking whether the specified foods were avoided in the dietary intake at the different study time points in group A and B regarding each food (**a**-**k**) during the examination dates (B) Baseline, (12 m) 12 months after loading, (24 m) 24 months after loading and (60 m) 60 months after loading. The comparison of the two groups at the individual time points took place with the exact test according to Fisher. (*p* ≤ 0.05; no correction of the p-value for multiple testing was performed); * *p* > 0.05. This was only significant (*p* = 0.002) for apples with peel at baseline

Significant intergroup differences between the two groups at the time of baseline were only evident for apples with peel. Whereby more subjects avoided apples in group A (*n* = 30; 63.8%), compared to group B (*n* = 14; 31.1%). However, over the 60 months after loading, this difference between the two study groups became non-significant. Similar results are observed for the remaining 10 foods. Again, no significant differences were observed between the two groups at any study times during the first 60 months. Figure 2 provides an overview of the different foods, asking whether the foods were avoided (answered “yes” in the questionnaire) or not (answered “no” in the questionnaire).

No significant intragroup differences over time were observed for seven of the 11 foods (sticky candy, lettuce, berries, grilled chicken, raw tomatoes, fried fish, brown bread). For the other four foods (apples with peel, whole nuts (not chopped), chops and steak, raw carrots), significant intragroup differences were observable over time, mainly in group A, leading to less avoidance of these foods. The consumption of whole nuts (*p* = 0.035) and raw carrots (*p* = 0.021) demonstrated a significant increase in group B between baseline and 60 months follow-up. An overview of the intergroup an intragroup comparison is given in Table 6.

**Table 6:** Comparison of time course regarding the avoidance habits towards the specified food within the two groups a and B for differences using the Cochrane-Q-test (*p* ≤ 0.05; no correction of the p-value for multiple testing was performed). Pairwise comparison of the baseline investigation to each study times for groups a and B using the McNemar-Test. (*p* ≤ 0.05; no correction of the p-value for multiple testing was performed); * *p* > 0.05

Foods	Comparison of time course regarding the avoidance habits towards the specified food		Baseline vs. 12 months after loading		Baseline vs. 24 months after loading		Baseline vs. 60 months after loading	
	Group A	Group B	Group A	Group B	Group A	Group B	Group A	Group B
Apples with peel	*p* = 0.001	*	*p* = 0.001	*	*p* = 0.002	*	*p* = 0.008	*
Whole nuts, not chopped	*p* = 0.028	*	*	*	*p* = 0.035	*	*p* = 0.019	*p* = 0.035
Sticky candy	*	*	*	*	*	*	*	*
Raw carrots	*p* = 0.040	*	*p* = 0.041	*	*	*	*p* = 0.041	*p* = 0.021
Chops or steaks	*p* = 0.005	*	*p* = 0.021	*	*	*	*p* = 0.002	*
Lettuce	*	*	*	*	*	*	*	*
Berries (strawberries, raspberries, blueberries, etc.)	*	*	*	*	*	*	*	*
Grilled chicken	*	*	*	*	*	*	*	*
Raw tomatoes	*	*	*	*	*	*	*	*
Fried fish	*	*	*	*	*	*	*	*
Dark bread	*	*	*	*	*	*	*	*

## Discussion

The present study investigated the influence of the loading protocol of implants placed in the midline of edentulous mandibles on different implant- and patient-related parameters. Here, the patients’ dietary habits were analyzed. A questionnaire was used to evaluate the subjective nutritional intake of patients at different times in the study. The methods used in this investigation were developed following already established procedures and were adapted to the presented study population and the socioeconomic and cultural environment of the patients [46, 47].

Midline implants in the mandible to support complete dentures have already been successfully investigated in the literature. They are characterized by the fact that an increase in chewing ability and patient satisfaction can be achieved with little effort and at minimal financial cost. While the performance in comparison to conventional [17, 18] and implant-supported prostheses on two implants [28, 29] has also been investigated, there is little evidence regarding different loading protocols of the implants. Therefore, the aim of the present study was to investigate the influence of direct or delayed loading of midline implants in the mandible.

In both investigation groups, ball attachments were used to anchor the dentures to the implants so that a comparison between the groups was possible. While under clinical conditions, ball attachments and locators show comparable satisfactory results in terms of chewing ability and prosthetic success when retaining mandibular overdentures on single midline implants [48, 49], ball attachments show a significantly better and extended retentive capability under in-vitro conditions. The present system uses metal matrices that are less affected by wear [50]. The retention can be adapted to the patient’s needs and demands throughout the patient’s life thanks to the possibility of switching to inserts of different strengths [51].

While in the first part of the questionnaire, a quantitative estimation of consumed amounts was chosen to describe dietary habits [45], in the second part active avoidance of certain foods was performed by answering simple “yes” and “no” questions [46]. In addition to the different nutrients, the food items queried in the questionnaires also include different textures and consistencies. As a result, the chewing function and the dental prosthesis are challenged in different ways. Depending on the specific food, bite-off functions (e.g. apples with peel) are performed by the anterior teeth and grinding processes of foods with different textures and consistencies (e.g. leaf vegetables, grilled chicken, grilled meat) are performed by the posterior teeth. At the same time, the foods also represent different challenges for the dentures: sticky candies can thereby dislodge the prosthesis during mastication, and small components such as grains or berries can in turn get under the prosthesis [47]. The objective chewing efficiency of the study population investigated here has already been presented by Passia et al. [34]. In both study groups, objective chewing efficiency was significantly increased already 4 months after loading of the implants and no influence of the loading protocol could be detected here. This significant improvement continued to be demonstrated after 60 months compared to baseline [34, 36]. At the same time, the implant-supported prostheses were evaluated by the patients with a significant improvement in the parameters comfort, function, stability and overall already after four months. Thereby, the delayed loading showed significant advantages regarding comfort and stability [37]. In the first week after implantation, patients in the delayed loading group showed significantly less postoperative pain and swelling than patients with immediate loading [38]. In both groups, the conditions for an improved nutrition intake even with challenging foods were created. Comparing the subjective evaluation of chewing capacity by the patients, no change in dietary habits could be noted despite an improved chewing function.

When comparing the documented dietary habits of both groups between baseline and 60 months follow-up, only in group A a significant improvement (*p* = 0.022) could be observed in the consumption of raw food. For all other foods, no change was observed in any of the groups. Although there were a few significant changes in individual foods in both groups when comparing baseline to the 12- and 24-month examinations, no significant change in dietary habits was permanently observed after 60 months of observation. This reveals that even the initial improvement in masticatory efficiency within the first few months had no effect on the patients’ nutrition. The influence of the time of loading after 60 months of follow-up was only significant for fresh fruit (*p* = 0.045) in favor of group B. However, since this difference had already been significant at baseline, it indicates that there was no change in dietary habits over the entire study period either. The fact that increased chewing efficiency is not accompanied by a change in nutrition intake is in accordance to previous results published in the literature. Nutrition appears to be dependent on many different factors, whereby chewing efficiency only plays a minor role. Individual preferences, personal tastes, social habits, religious environment, economic status, and general health of patients also have a sustained influence on diet [52–55]. In addition, age-related aspects can also have a major influence on dietary habits. Besides physiological changes, such as low mobility or reduced sensitivity in geriatric patients, this can also be caused by taking medication. In turn, these can lead to changes in taste or a reduced flow of saliva, which in turn restricts food intake, mastication and digestion [56–58]. Alcoholic beverages also seem to be very dependent on individual tastes, which is why there have been no changes in consumption here either. A limitation of the present study is that patient-specific preferences and/or aversions towards the consumption of certain foods as well as the reason for a possible avoidance were not included in the questionnaire. For example, foods that were already avoided at baseline due to individual tastes would also be avoided after implantation, which may bias the results. Ettinger [59] concluded that the use of implants can measurably increase chewing efficiency compared with conventional prostheses. However, he proclaimed that this change on its own did not motivate changes in nutritional intake. For this purpose, patients should receive concomitant interdisciplinary nutritional counseling [19, 55, 59]. Studies on the treatment of edentulous patients with conventional full dentures in combination with nutritional counseling show positive changes with regard to a change in diet [60, 61]. While a change does not only result from the increased chewing efficiency, it can help patients to consume certain foods again that had become impossible to masticate as a result of a poorly fitting prosthesis [12]. This is also evident in our investigation when looking at the results of the second part of the dietary questionnaire. The active avoidance in the consumption of food due to the dental prosthesis was queried. Foods that were initially avoided very strongly (over 80%, sticky sweets) or significantly less (under 25%) did not show any significant change in the further follow-up examinations, regardless of the loading protocol. However, the group of foods that were often avoided (over 50%; apples with peel, whole nuts, raw carrots, chops and steaks) and had a hard or rough consistency showed a significant potential for improvement. It should be added, that in group B, at baseline, apples with peel had already been avoided significantly less, and due to the improvement in group A, the avoidance behavior at the post-testing time points was equalized. For all four foods, a significant improvement was observed after 60 months in the group of immediate loading. In the group of delayed loading for at least two foods (whole nuts and raw carrots), an improvement was found. While in group B this change could only be noticed after 60 months, in group A a significant improvement could already be observed after 12 and 24 months. This suggests that the intake of foods that are often avoided actively due to their texture or consistency can be improved, whereas foods that can cause the prosthesis to lift off or proportionally come under the prosthesis reveal no change. A similar behavior could be observed by Quandt et al. [46]. In the present study, it was not recorded, in which form and with which procedures the foods were processed, which could lead to changes in size and texture.

The present investigation did not demonstrate any significant changes in dietary habits as a function of the loading protocol. The nutritional intake of patients before implant placement was documented as a reference point for the present clinical study. At this time, the existing complete prostheses in the mandible and maxilla had been in situ for at least three months. In order to obtain comparative times, the nutritional intake of the patients was surveyed again at 12, 24 and 60 months after loading of the implants. Whereas in case of delayed loading, direct anchorage of the prosthesis to the implant took place after a defined healing period of 3 months, in the case of immediate loading of the implants, the loading time corresponds to the implantation time [21]. The lack of a healing time in the immediate loading group means that the direct benefit of the therapy can be obtained at an earlier stage [62]. When considering the parameters comfort, function, stability/fit of the mandibular prosthesis as well as speech and overall evaluation, the immediate loading group showed significantly better values four weeks after implant placement than the delayed loading group [36]. This is probably due to the fact that the benefit of the implants had not yet been realized in the delayed loading group due to a still absent anchorage of the prosthesis. The healing phase in the delayed loading group seems to have an important role, which should be interpreted positively for the implants with immediate loading. The aspect of a possible advantage in the immediate loading group due to the lack of a healing phase cannot be evaluated with the present study design. In this case, the study periods for this parameter had to be selected depending on the time of implant placement rather than the time of loading.

## Conclusion

Under the limitations of the presented study, the results demonstrate that the insertion of a single midline mandible implant for fixation of a mandibular complete denture is not expected to result in permanent significant changes in the patients’ dietary intake, regardless of the loading protocol of the implants. However, the investigation reveals that foods that are often avoided and have a hard and coarse texture are significantly less avoided after 60 months. Thus, a change in diet seems possible in purely theoretical terms.

Considering a possible impact of the loading protocol, the examination times should be selected depending on the implant insertion, since the healing phase of the delayed loading or the absence of this phase in the context of immediate loading should also be taken into consideration.

## Data Availability

No datasets were generated or analysed during the current study.
